# Simulation of Stimulation: Cytokine Dosage and Cell Cycle Crosstalk Driving Timing-Dependent T Cell Differentiation

**DOI:** 10.3389/fphys.2018.00879

**Published:** 2018-08-02

**Authors:** Matteo Barberis, Tomáš Helikar, Paul Verbruggen

**Affiliations:** ^1^Synthetic Systems Biology and Nuclear Organization, Swammerdam Institute for Life Sciences, University of Amsterdam, Amsterdam, Netherlands; ^2^Molecular Cell Physiology, VU University Amsterdam, Amsterdam, Netherlands; ^3^Department of Biochemistry, University of Nebraska-Lincoln, Lincoln, NE, United States

**Keywords:** cytokine dosage, cytokine activation timing, (memory) T cell differentiation, cell cycle, CDK, p27^Kip1^, autoimmunity, MAmTOW

## Abstract

Triggering an appropriate protective response against invading agents is crucial to the effectiveness of human innate and adaptive immunity. Pathogen recognition and elimination requires integration of a myriad of signals from many different immune cells. For example, T cell functioning is not qualitatively, but quantitatively determined by cellular and humoral signals. Tipping the balance of signals, such that one of these is favored or gains advantage on another one, may impact the plasticity of T cells. This may lead to switching their phenotypes and, ultimately, modulating the balance between proliferating and memory T cells to sustain an appropriate immune response. We hypothesize that, similar to other intracellular processes such as the cell cycle, the process of T cell differentiation is the result of: (*i*) pleiotropy (pattern) and (*ii*) magnitude (dosage/concentration) of input signals, as well as (*iii*) their timing and duration. That is, a flexible, yet robust immune response upon recognition of the pathogen may result from the integration of signals at the right dosage and timing. To investigate and understand how system’s properties such as T cell plasticity and T cell-mediated robust response arise from the interplay between these signals, the use of experimental toolboxes that modulate immune proteins may be explored. Currently available methodologies to engineer T cells and a recently devised strategy to measure protein dosage may be employed to precisely determine, for example, the expression of transcription factors responsible for T cell differentiation into various subtypes. Thus, the immune response may be systematically investigated quantitatively. Here, we provide a perspective of how pattern, dosage and timing of specific signals, called interleukins, may influence T cell activation and differentiation during the course of the immune response. We further propose that interleukins alone cannot explain the phenotype variability observed in T cells. Specifically, we provide evidence that the dosage of intercellular components of both the immune system and the cell cycle regulating cell proliferation may contribute to T cell activation, differentiation, as well as T cell memory formation and maintenance. Altogether, we envision that a qualitative (pattern) and quantitative (dosage) crosstalk between the extracellular milieu and intracellular proteins leads to T cell plasticity and robustness. The understanding of this complex interplay is crucial to predict and prevent scenarios where tipping the balance of signals may be compromised, such as in autoimmunity.

## Introduction

Selecting the appropriate responses against invading agents is crucial to the effectiveness of the human immune system. It has to be simultaneously flexible, i.e., able to recognize all possible threats and robust to prevent a too vigorous or unnecessary response. Its relevance is especially pronounced in individuals exhibiting genetic or acquired immune deficiencies, which result in extreme sensitivity to pathogen invasions that may lead to severe pathology, or death.

The immune system can be divided in two sub-systems: the innate, or non-specific immune system, and the adaptive (acquired), or specific immune system. The former serves as a quick-acting defense line that promptly identifies and eliminates potentially harmful foreign or endogenous particles. It consists of: (*i*) specialized cells, such as natural killer (NK) cells, a type of cytotoxic lymphocytes that function similarly to cytotoxic T cells, by rapidly responding to virus, infected cells, and phagocytes, cells that ingest harmful foreign particles, and (*ii*) the humoral complement system, which consists of macromolecules found in extracellular fluids such as complement proteins and secreted antibodies, and the interferon system present in all nucleated cells, including the ones of the immune system.

Conversely, the adaptive immune system uniquely targets and destroys invading pathogens and includes T and B lymphocytes. T (thymus) cells are involved in cell-mediated immunity, whereas B (bone marrow) cells are crucial for adaptive humoral immunity, leading to the production of large amounts of antibodies. These cells specifically recognize “non-self” antigens during a process known as antigen presentation mediated by antigen-presenting cells (APCs), such as dendritic cells and macrophages. Upon a pathogen attack, T_h_ (helper) cells produce small molecules called cytokines, among which chemokines and interleukins, whereas T_c_ (cytotoxic) cells produce toxic granules that contain enzymes which kill pathogen-infected cells. Beside specificity, adaptive immunity also retains a memory of invading pathogens, for both T cells and B cells, whereas innate immunity does not.

The adaptive immune system protects mammals from a myriad of invading and potentially harmful agents such as bacteria, viruses and toxins. Dendritic cells, macrophages and other APCs transport encountered antigens to the thymus. Here, the foreign molecules are processed by a set of T (thymus) cells selected for recognizing a specific non-self antigen. If a successful match occurs, the antigen (together with other stimulatory factors present on and secreted by the APC) will lead to activation and proliferation of T cells with the corresponding specificity.

A naïve precursor T cell, also indicated as T_h_0 cell, is a T cell that has successfully undergone the selection process (licensing) in the thymus. Naïve T cells may differentiate into several lineages, based on the selection and shaping of an immune response most appropriate for the invading agent. T cell activation can occur by recognition of a single antigen independent of its maturation and differentiation state. Further, T cell activation depends on (*i*) the nature of the presented antigen, (*ii*) the presence and abundance of small proteins called cytokines (e.g., chemokines and interleukins), and (*iii*) APC-costimulatory signals, (Huang et al., 2013; Wertek and Xu, 2014). After activation, T cell differentiation into one of the various lineages is thought to result from the strength, duration and ratio of extracellular stimuli transmitted through the T cell receptor (TCR), which is responsible for the recognition of the presented antigen (Tubo and Jenkins, 2014), and through a number of other receptors sensing the cytokines.

**FIGURE 1:**
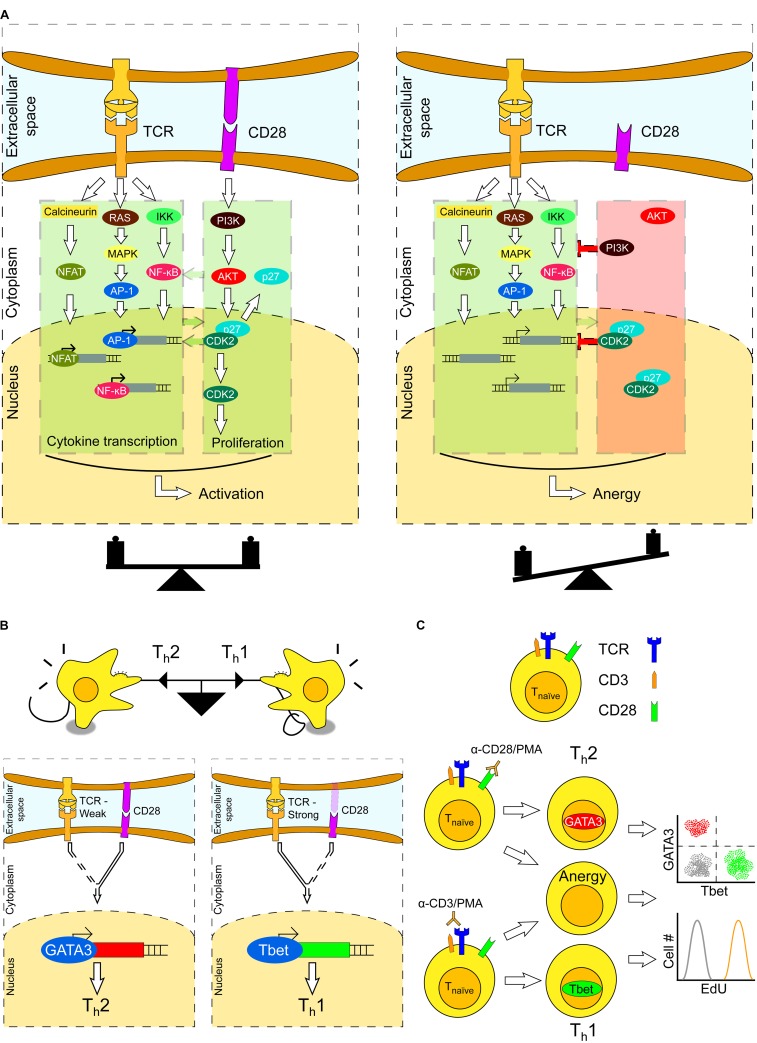
T cell fate upon TCR activation and CD28 stimulation. **(A)** Successful activation of a T cell occurs through the TCR signaling cascade and CD28 co-stimulation. Stimulation of both TCR and CD28 results in T cell activation (left), whereas stimulation of TCR without CD28 co-stimulation results in T-cell anergy (right). **(B)** T cells constantly integrate lineage-specific extracellular signals; the strongest signals eventually dictate a definite lineage a T cell will be committed to, in a tug-of-war game (upper). This balance is dictated by a trade-off-weight of lineage-specific transcription factors. For example, differentiation of T_h_ cells into T_h_1 or T_h_2 phenotypes is decided when either GATA-3 (T_h_2) or T-bet (T_h_1) is expressed (lower). Which of these transcription factors is expressed is the result of tipping the balance between factors that function upstream in the TCR and CD28 signaling cascades. The amount of these factors is dependent on the strength of TCR- and CD28-mediated stimulation upon antigen recognition. **(C)** Functional T cells are characterized by the T cell receptor (TCR), co-receptor CD3 and CD28 co-stimulation (upper). T cells can be activated and differentiated *in vitro* by crosslinking the TCR with CD3 antibodies and PMA treatment. Additionally, CD28 can be triggered by antibodies directed against it, mimicking APC stimulation. The activation/proliferation status can be monitored by single-cell analysis of lineage-specific transcription factors and DNA synthesis.

To unveil the protective potential of a T cell fully, its mere activation is not enough. Secondary proliferative signals are also required for a T cell to become fully engaged. Proliferation upon antigen recognition is mainly initiated by co-stimulation of CD28 (Cluster of Differentiation 28), expressed on the T cell surface, which is required for T cell survival. The co-stimulatory signals provided by CD28 lead to the production of T cell clones capable of recognizing the invading agent with high affinity and specificity (Boomer and Green, 2010; Esensten et al., 2016). As the added activation/proliferation signal is stronger in T cells with their TCR actively engaged, a bias is created toward survival of the T cells with the strongest affinity to the antigen. In **Figure 1A**, the intracellular signaling cascades triggered upon TCR activation and CD28 stimulation are illustrated as two separate pathways, although crosstalk between these pathways exists. In the figure, examples of this crosstalk have been highlighted by green arrows. Stimulation of CD28 leads to PI3K activation, as well as of several regulators of cell cycle progression, which in turn reinforces the strength of the TCR signaling cascade by further promoting stimulation of the Nuclear transcription Factor kappaB (NF-κB) (Beyersdorf et al., 2015). This regulates the expression of genes involved in inflammation and cell survival. Moreover, cyclin-dependent kinases (CDKs) have a role in the balance between T cell activation and anergy – “a tolerance mechanism in which a lymphocyte is functionally inactivated following the contact with an antigen, but remains alive for an extended period of time in a hyporesponsive state” (Schwartz, 2003). Specifically, CDK4 phosphorylates and enhances the activity of the AP-1 family of proteins (Vanden Bush and Bishop, 2011), which is critical in transcriptional regulation of T cells (see Li et al., 2014, for a recent review). Furthermore, CDK2 is necessary for normal T cell differentiation, as CDK2-deficient T cells exhibit an anergic state, even if CD28 co-stimulation is present (Chunder et al., 2012). Thus, T cell proliferative signals are essential for T cell survival, and reinforced by coupling cell cycle events occurring upon CD28 stimulation with TCR activation.

The (added) strength of both CD28 and TCR signals may have several consequences based on the modulation of the CD28/TCR balance: (*i*) the T cell will undergo proliferation when CD28 is activated together with TCR; (*ii*) the T cell will undergo anergy when CD28 is not activated, although TCR is fully active. In the former scenario, the T cell will develop into the so called T helper (T_h_) cells that express on their surface the glycoproteins CD4 (Cluster of Differentiation 4) or exhibit cytotoxicity when carrying CD8 (Cluster of Differentiation 8). After maturation of T cells, these may become CD4+ (helper, T_h_) or CD8+ (cytotoxic, T_c_) lineage of T cells (Germain, 2002), also known as effector T cells. CD4+ T cells recognize antigens presented on MHC (Major Histocompatibility Complex) class II molecules by APCs, such as dendritic cells, and regulate the immune response by releasing specific cytokines. Conversely, CD8+ T cells recognize antigens presented on MHC-class-I molecules, and release cytotoxic molecules, such as perforins, that compromise membrane integrity and/or enter the cytoplasm of damaged (cancer, infected) cells triggering apoptosis.

How the activation and balance of TCR and CD28 pathways contribute quantitatively to T cell activation and lineage decision has not been conclusively investigated in *in vivo* settings. Furthermore, *in vitro* experiments have to rely on administration of activating reagents and/or stimuli, or inhibitory agents (e.g., the PI3K inhibitor Wortmannin) to stimulate the activity of intracellular factors. For example, T cell differentiation protocols rely on specific antibodies as stimuli for TCR and/or CD28 for either a limited temporal window or throughout the entire course of the experiment (Avni et al., 2002; Flaherty and Reynolds, 2015). Therefore, setting the level of activation is the result of stability, availability, and concentration of multiple reagents/antibodies.

Upon stimulation of TCR and CD28, the respective downstream signaling cascades are activated in a process for a T cell to proliferate. However, any perturbation in the amounts of the cytokines responsible for TCR and CD28 activation may tip the balance shown in **Figure 1A** from T cell activation to anergy. For this delicate balance to hold, thereby for T cell proliferation to occur, cytokines produced in the environment shall be present at definite levels and ratios. Although the type and abundance of cytokines have been shown to induce specific T cell fates (Rowbottom et al., 1999; Jones, 2005; Kimura and Kishimoto, 2010; Read et al., 2016; Eizenberg-Magar et al., 2017; Kaartinen et al., 2017), *precise* levels at which cytokines are required have not been determined yet.

Recently, we have devised a methodology to determine quantitatively the effects of gene dosage, thereby protein concentration, on *in vivo* cellular integrity, providing a detailed example for the eukaryotic cell cycle (Barberis and Verbruggen, 2017). This methodology, which we coined “Maximum Allowable mammalian Trade-Off-Weight” (MAmTOW), relies on gene engineering strategies, such as the CRISPR/Cas9 technology, and may be combined with optogenetic tools that enable – upon light induction – the nuclear import and export of tagged proteins. The goal of the methodology is to achieve a *precise* measurement of upper limit gene copy number (gene dosage) and microscopy-based visualization of protein spatiotemporal localization. Integrating this output with computer models provides information on cellular robustness (Barberis and Verbruggen, 2017). Here, we propose that genetic engineering technologies such as the MAmTOW may also be successfully employed to investigate the weight of individual cytokines as well as components of TCR and CD28 pathways to tip the balance that modulates T cell activation, lineage decision and plasticity.

## Cytokine Pattern and Dosage Determine T Cell Differentiation

The fate of T cells relies on TCR activation and on the presence and abundance of specific cytokines. Several studies have shown that the concentration of a single cytokine, for instance an interleukin, can influence the outcome of T cell activation and proliferation *in vitro*. For example, IL-10 inhibits the activation of CD8+ T cells in a dose-dependent manner (Rowbottom et al., 1999). By way of another example, IL-6 modulates the balance between regulatory T cells (also indicated as Treg) and T helper 17 cells (also indicated as Th17), which are CD4+ T cells involved in the prevention and (pro-inflammatory) induction of autoimmune diseases, such as rheumatoid arthritis. In this context, IL-6 promotes Th17 development from a naïve T cell, but inhibits Treg differentiation (Kimura and Kishimoto, 2010). Low IL-2 concentrations favor the formation and proliferation of memory T cells (Kaartinen et al., 2017), as well as modulating the balance between Treg and Th17. The disruption of this balance is characteristic of autoimmune diseases (Kosmaczewska, 2014). Interestingly, IL-6 and IL-2 are crucial proliferation and differentiation stimuli, and their effects are both pleiotropic and ambiguous depending on their abundance, ratio and T cell type they act upon (Jones, 2005; Read et al., 2016). Altogether, this evidence indicates that it is not the presence or absence of specific cytokines that determines the fate of T cells, but their *precise* dosage (concentration). To understand the relevance of cytokines for T cell differentiation, qualitative information is therefore insufficient, whereas quantitative information of cytokine action is desired.

The mode(s) of action of cytokines is (are) highly context- (and timing-) dependent. For example, as compared to the T cells that actively respond to a stimulus and induce some changes in the immune response, memory T cells have encountered, and responded to, their cognate antigen during a prior exposure to a pathogen. When a second exposure occurs, memory T cells recognize the invaders and initiate a much faster and stronger immune response as compared to their naïve counterparts. For example, the timing of stimulation of the first generation of primary memory CD8+ T cells increases the responsiveness of the second generation of memory CD8+ T cells (Khan et al., 2015).

In a recent publication by Eizenberg-Magar et al. (2017), a large repertoire of input cytokines was tested to investigate differentiation of CD4+ T cells. By measuring the expression of four important lineage-specifying transcription factors, i.e., GATA-3, T-bet, Foxp3, and RORγt as a function of the type of input cytokines administered, the study has revealed that a number of varied input cytokine combinations determines CD4+ T cell differentiation. Strikingly, CD4+ T cells do not just exhibit the discrete canonical phenotypes described in literature, but also show a wide range of intermediate differentiation states (Eizenberg-Magar et al., 2017). However, this work considers different cytokine patterns and does not investigate the importance of the interleukin concentrations relative to each other, for the occurrence of these intermediate phenotypes.

Here, we propose that both a relative interleukin dosage and a discrete interleukin pattern may specify the appearance of T cell phenotypes. Currently, it is a challenge to investigate the interplay, both qualitative (*pattern*) and quantitative (*dosage*), between the interleukins required for T cell differentiation, both *in vitro* and *in vivo*. This is because the interplay appears to be dependent on a number of variables difficult to control, such as the stochastic expression of the TCR and cytokine receptors, leading to cell-to-cell variability in the strength of the received signal(s).

In such a precision-exalting scenario, a systems biology analysis may provide support to understand the complex dynamics underlying T cell differentiation. Specifically, to investigate the formation of all possible T cell phenotypes at a systems level, integration of high-quality and, most importantly, *precise* experimental analyses, such those presented by Eizenberg-Magar et al. (2017), with sophisticated computer models is of vital importance. Computer models of T cell differentiation can yield predictions on the specific phenotypic lineage decision by varying the pattern and/or dosage of cytokines – including, but not limited to, interleukins – which can be tested experimentally.

While a number of computer modeling approaches are available, T cell differentiation has been investigated by employing at least three complementary strategies. First, the model of Eizenberg-Magar et al. (2017) is considered as a “black box,” and represented as a linear regression model. This model was used to characterize the input–output relationships between the patterns of T cell stimuli and the production of their characteristic cytokines. While the construction and analysis of such statistical models can be efficient, regulatory information about the components of the biochemical networks that mediate the T cell differentiation process is lacking. Hence, these approaches are unable to be employed to further investigate molecular mechanisms driving the process. This is where mechanistic computational models become useful. Such models take into account the specific (and detailed) mechanisms of regulation of the components (genes, proteins, regulatory molecules, etc.) considered. These models can be represented with a plethora of mathematical approaches (many are reviewed in Le Novère, 2015), including kinetic, or Ordinary Differential Equations (ODEs), and logical modeling, which have been applied to address T cell differentiation.

Kinetic models offer the advantage to incorporate specific kinetic parameters (known and/or estimated) to represent dynamics of components. For example, an ODE model of Carbo et al. (2013) is generated to simulate T cell differentiation to predict, and subsequently validate *in vivo*, the role of peroxisome proliferator-activated receptor-gamma (PPARγ) as a modulator of the switch between T_h_17 and inducible Treg phenotypes. In another study, the same authors have developed an ODE model to predict and provide basis for an *in vivo* validation that IL-21 regulates T_h_1 and T_h_17 responses during chronic *Helicobacter pylori* infection (Carbo et al., 2014b). On the other hand, logical models provide a (kinetic) parameter-independent approach to address the dynamics of (large-scale) models. These have been employed to investigate the regulatory network controlling the differentiation of Th cells (Mendoza, 2006), as well as plasticity of differentiated CD4+ T cells (Naldi et al., 2010; Abou-Jaoudé et al., 2015; Martinez-Sanchez et al., 2015). Computational modeling has helped to advance the understanding of CD4+ T cell plasticity and function, and existing as well as novel computational strategies have been recently summarized (Carbo et al., 2014a).

To aid the generation, validation and refinement of such computer models, here, we propose a strategy to retrieve quantitative information on immune response progression. Importantly, we hypothesize that, similar to other intracellular processes such as the cell cycle, the process of T cell differentiation is the result of: (*i*) pleiotropy (pattern) and (*ii*) magnitude (dosage/concentration) of input signals, as well as (*iii*) timing and duration of their emergence and stability. We will first develop these concepts on the activation of CD4+ T cells and their subsequent proliferation and differentiation. Subsequently, we will speculate about possibilities to investigate their development into memory T cells. Finally, we will envision a strategy to investigate how components of the signaling cascade leading to the immune response and those of cell cycle progression may crosstalk to impact timing of the immune response. To rationalize the latter point, we will propose dedicated experiments, based on genetic engineering technologies, to alter the dosage of a pivotal cell cycle timer, the CDK inhibitor p27^Kip1^ (p27), and the CD28 cytokine receptor.

## A Requirement of Dose-Dependent Stimuli for T Helper Cell Decision Making

Cluster of Differentiation 3 (CD3) is a TCR co-receptor and contributes to the activation of CD4+/CD8+ T cells. Cross-linking of TCR by anti-CD3 antibodies (TCR/CD3 stimulation) elicits a strong activation of downstream intracellular signaling cascades (Dave, 2009; Smith-Garvin et al., 2009). Together with TCR/CD3 dimerization, CD28 is also required for T cell survival, and absence of CD28 co-stimulation results in T cells to remain in an anti-proliferative, or anergic, state (**Figure 1A**).

CD4+ T cells mainly differentiate to T helper (T_h_) cells. These contribute to the immune response by helping maturation of B cells into plasma cells and memory B cells, as well as by activating CD8+ T cells, or cytotoxic (T_c_) cells. Depending on the nature of stimulatory signals encountered, naïve CD4+ T cells can differentiate into any of a number of phenotypes, such as T_h_1, T_h_2, T_h_17, and Treg cells (O’Shea and Paul, 2010; Zhu et al., 2010). Once fully activated, these cells divide rapidly and secrete different cytokines that regulate different types of immune responses. The major phenotypes into which naïve CD4+ T cell differentiate are T_h_1 and T_h_2, which serve as effectors against intracellular and extracellular invaders, respectively. The balance between these two cell types may be monitored through the relative abundance of two lineage-specific transcription factors: T-bet (leading to the T_h_1 phenotype) and GATA-3 (leading to the T_h_2 phenotype) (Murphy and Stockinger, 2010). Absence of any of these two transcription factors, due to the absence of the CD28 co-stimulatory signal, does identify non-responding, anergic cells.

Triggering of the CD28 co-receptor in either absence of a TCR ligand or presence of a weak ligand stimulating the TCR yields a predominant T_h_2 phenotype. Conversely, a strong TCR stimulation with a reduced (but not absent) CD28 stimulus results in a T_h_1 phenotype (**Figure 1B**) (Riha and Rudd, 2010). A method to selectively trigger a T_h_1 or T_h_2-specific response *in vitro* has been described (Smeets et al., 2012), which relies on stimulation of cells with Phorbol 12-myristate 13-acetate (PMA) and antibodies specific to either CD3 or CD28. Stimulation with PMA/CD3 antibodies triggers a T_h_1 response, whereas stimulation with PMA/CD28 yields a T_h_2 response. Considering that in neither case both stimuli shall be present, a significant fraction of treated cells will remain non-responsive or become anergic. The stimulation scheme and the possible outcomes are displayed in **Figure 1C**. By measuring the expression level of T_h_1/T_h_2 lineage-specific transcription factors and BrdU/EdU incorporation, the differentiation and proliferation state may be determined.

Within this scenario, an interesting aspect is represented by the non-anergic T cell population. Why is a small fraction of cells activated, whereas the large majority is not? The question cannot be satisfactorily answered by considering exclusively external factors as, in principle, one triggering event is sufficient for T cell activation. Furthermore, memory T cells respond faster to antigen recognition as compared to naïve T cells, thus reinforcing the idea that extracellular stimuli cannot be the only signals responsible for differences in the pattern of phenotypes appearing upon T cell activation (Cuddapah et al., 2010). For example, poised RNA polymerase II has been suggested to control fast induction kinetics of several cytokines, and to contribute to the swift response capacity of memory T cells, putatively explaining fast response of one T cell phenotype but not others (Adelman et al., 2009; Cuddapah et al., 2010). Furthermore, cell-to-cell variability upon naïve T cell activation may be explained by chromatin permissibility for transcription of definite activation regulators (Mariani et al., 2010). These mechanisms have been described for specific subtypes of T cells, and may be employed by the immune system to fine-tune the quality of the response upon encountering an antigen. However, a general control system that may rationalize the consequential appearance of the various T cell phenotypes – if at all existing – has not been elucidated yet.

Here, we propose that a less specific, yet effective dynamic regulation may be in place to control the pattern of T cell phenotypes, which is directly dependent on the timing at which such phenotypes appear upon T cell activation. That is, variability in the abundance of both TCR and CD28 signaling cascades may play a role in T cell activation and differentiation dynamics, as well as in the timing of the immune response upon recognition of the invader. Example of targets that are activated upon TCR- and CD28-mediated stimulations, and that may be tested for their relevance in T cell differentiation dynamics, are the ZAP/70 kinase and its substrates, LAT adaptor (membrane-bound) and SLP-76 adaptor (soluble) proteins, downstream the TCR cascade (Pennock et al., 2013; Ngoenkam et al., 2017), as well as the PI3K kinase and the IP3 secondary messenger molecule downstream the CD28 cascade (Bjørgo and Taskén, 2010). Understanding of the relevance of dosage and timing of input signal for T cell activation and differentiation can be nowadays experimentally investigated by CRISPR/Cas9-based approaches such as the methodology that we have recently envisioned (Barberis and Verbruggen, 2017).

A recent paper describes how TCR signaling can be studied by CRISPR/Cas-9 technology in immortalized Jurkat T cells (Chi et al., 2016). We provide here an example of how this technology can be of use based on a modification of our earlier devised MAmTOW strategy applied to these cells. This strategy uniquely describes the functioning of a synthetic cassette – consisting of a tetracycline repressor and a reported gene unit encoding a fluorescent protein (e.g., GFP) – integrated into the genome of mammalian cell lines, such as Jurkat T cells, which replaces the endogenous promoter of a target gene to allow for its tunable expression and quantification (Barberis and Verbruggen, 2017). Combining CRISPR/Cas-9 and MAmTOW systems would enable the replacement of components of the endogenous T cell signaling by tunable, engineered ones. Specifically, the aforementioned synthetic cassette may be engineered in Jurkat T cells, with the replacement alleles containing (*i*) an inducible tetracycline promoter that allows for a tunable expression of components, and (*ii*) a fluorescent tag to allow for an easy and high-throughput screening of their abundance (dosage). Thus, by adding tetracycline, the dosage of signaling components of choice can be modulated, and the (upper and lower) thresholds required for T cell activation and differentiation may be established quantitatively.

Through such as strategy, the experiments performed in the work of Eizenberg-Magar et al. (2017), where combinations of input cytokines result in the formation of a pattern of different CD4+ T cell phenotypes, may be conducted at a large scale by testing a number of signaling molecules by combining CRISPR/Cas-9 and MAmTOW methodologies. That is, by engineering T cells, the differentiation path as a function of both interleukin pattern/dosage and dosage of intracellular components may be determined. Thus, the effects of triggering TCR and CD28 simultaneously, or separately, may be investigated (**Figure 1C**, bottom left). By quantitative immunostaining or tagging of the transcription factors responsible for the differentiation state of T cells, e.g., T-bet and GATA-3, their expression can be concomitantly monitored to investigate T cell’s lineage commitment (**Figure 1C**, bottom right). Interestingly, a systems biology approach that integrates computer modeling and single-cell measurements was able to reveal that endogenous variation in the expression levels of signaling proteins affects antigen responsiveness during T cell activation, thereby influencing the phenotypic variability of cells (Feinerman et al., 2008). Hence, it is plausible to envision that a variable dosage of extracellular as well as intracellular stimuli influences T cell differentiation, thereby inducing the pattern of phenotypes that appear during the lineage decision process.

In the following sections, we will further elaborate on the relevance of determining dosage, expression limits and timing of intracellular signals upon TCR activation. We will rationalize possible scenarios of tipping the balance among signals, such that one of these may be favored or gains advantage on another one, and how they can be crucial to understand the underlying fundamentals of T cell activation and differentiation versus anergy. A comparison of those scenarios with an emphasis on T cell plasticity and reversibility of T cell differentiation phenotypes will be also presented. Lastly, we will propose a strategy to investigate memory T cell formation quantitatively, and suggest possible links between T cell differentiation and expression of cell cycle regulators.

## *Precise* Cytokine Dosage and Activation Timing Impact T Cell Lineage Decision

It is currently not understood how the cytokine pattern influences T cell fate both quantitatively (*dosage*) and temporally (*over time*). This paradigm has been not addressed yet in the field; however, the fundaments have been set by recent data showing that combining various cytokines resulted in the formation of a pattern of CD4+ T cell phenotypes (Eizenberg-Magar et al., 2017).

During the course of an immune response the amount of cytokines may vary for several reasons, such as for example antigen level, efficiency of antigen recognition by the T cells, or the number of T cells activated. Thus, we envision that definite cytokines produced during the immune response may be required in *precise* amounts, in order for a specific T cell phenotype to appear over time. Furthermore, we predict that the specific time at which cytokines are produced during the immune response determines the timing at which a T cell phenotype appears. A specific cytokine pattern may not suffice to promote a T cell response if the amount and ratio of cytokines are not appropriate. Hence, we propose that both individual cytokine dosage and relative dosage as well as their timing of activity, together with the cytokine pattern, are required for the appearance of a specific T cell phenotype at a definite timing. In support to this concept, it has been shown that the timing post-infection of the cytokine type I interferon (IFN) administration to CD8+ T cells with respect to TCR engagement by an antigen determines whether a T cell is either activated or triggered toward apoptosis (Crouse et al., 2015). This may be a concept that could be generalized also for the CD4+ T cell lineage differentiation.

**FIGURE 2:**
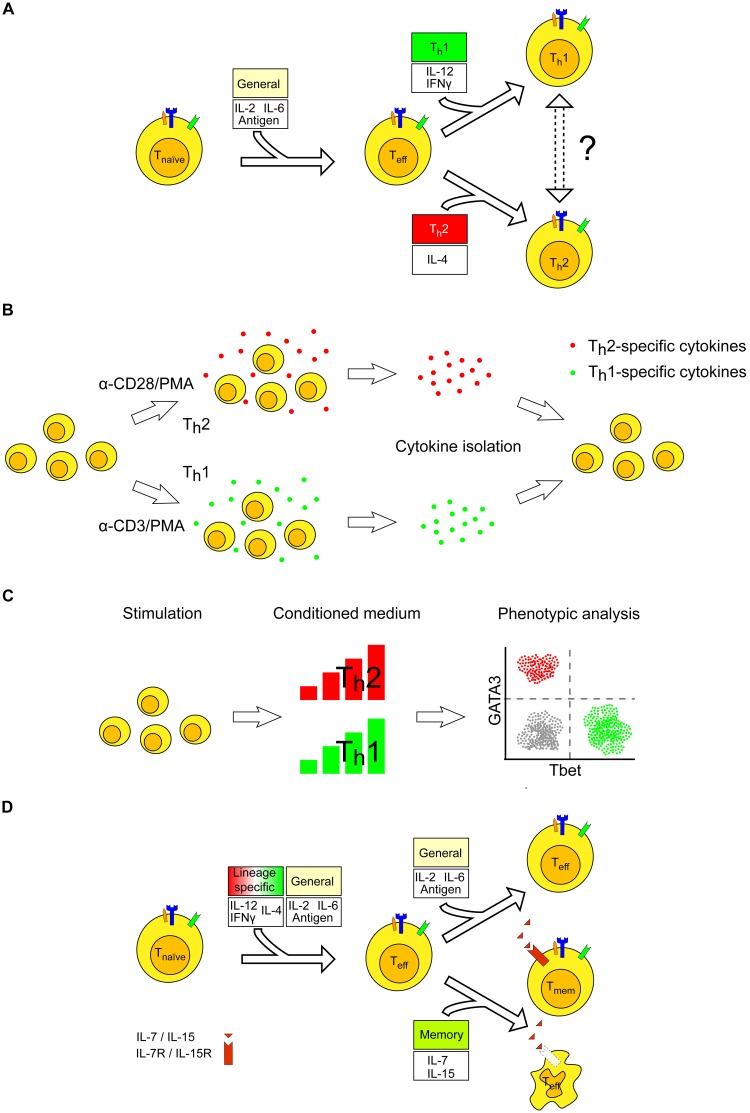
Experimental design for establishing cytokine requirement for differentiation to effector and memory T cells. **(A)** Several cytokines are involved in the balance dictating T effector cell activation, which ultimately results in a lineage commitment toward T_h_1 or T_h_2 phenotypes. After differentiation, T cells may exhibit plasticity, resulting in the ability to switch lineage commitment (dashed arrows) or to exhibit an intermediate phenotype (not shown). **(B)** T cells are stimulated with antibodies triggering CD3 or CD28 to cause a skew either toward the T_h_1- or T_h_2-specific lineages. The medium may then be added to naïve T cells to investigate whether the specific cytokines released may impact the T cell phenotype. **(C)** Similar experiments as in **(B)** can be performed by adding different doses of conditioned medium. Phenotypic analysis for GATA-3 and T-bet reveals how T cells have differentiated *in vitro*. **(D)** T cells require a constant activation of their TCR as well as of the appropriate pattern of cytokines to remain fully engaged during the process of antigen recognition. If the TCR is no longer activated, for example due to loss of antigen, T cells may either undergo apoptosis or differentiate to memory T cells. The latter requires stimulation by the survival signals IL-7 and IL-15. Typically, T cells undergoing apoptosis after an acute inflammation are less sensitive to these interleukins as compared to T cells that develop into memory T cells. Effector T cells are assumed to have their TCR engaged, but both antigen and APC are not displayed for sake of clarity.

The cytokine patterns required to generate the various canonical T cell phenotypes from a naïve CD4+ T cell are relatively well characterized. For example, the major phenotypes of T cell differentiation, T_h_1 and T_h_2, are triggered by IL-12 and IFNγ (T_h_1), and by IL-4 (T_h_2) abundance, respectively (**Figure 2A**). It may be possible to investigate whether the pattern of cytokines stimulating or produced by T_h_1 cells would be able to influence the T_h_2 lineage when these are introduced in *in vitro* cultures. In this scenario, conditioned supernatants derived from both T_h_1 and T_h_2 cells may be mixed in different ratios and added to a freshly activated naïve T cell at different times, in order to investigate the proliferative capacity and influence on the T cell lineage of specific cytokines (**Figure 2B**). Furthermore, a complementary scenario may be explored. Specifically, it can be investigated whether a definite lineage, for example a T_h_2-differentiated cell, would be able to revert its phenotype, becoming a T_h_1 cell upon expression of a variable dosage, and of variable timing of activity, of the cytokines that stimulate the appearance of the T_h_1 lineage. This plasticity has been recently shown, with IFNγ inducing T-bet expression and reprogramming T_h_2 cells upon viral infection. As reprogramming cannot occur in the absence of TCR stimulus *in vitro*, it may be concluded that cytokines alone are not sufficient to induce this plasticity (Hegazy et al., 2010). Similar scenarios have been observed also for other T cell subtypes (Hirahara et al., 2013).

**FIGURE 3:**
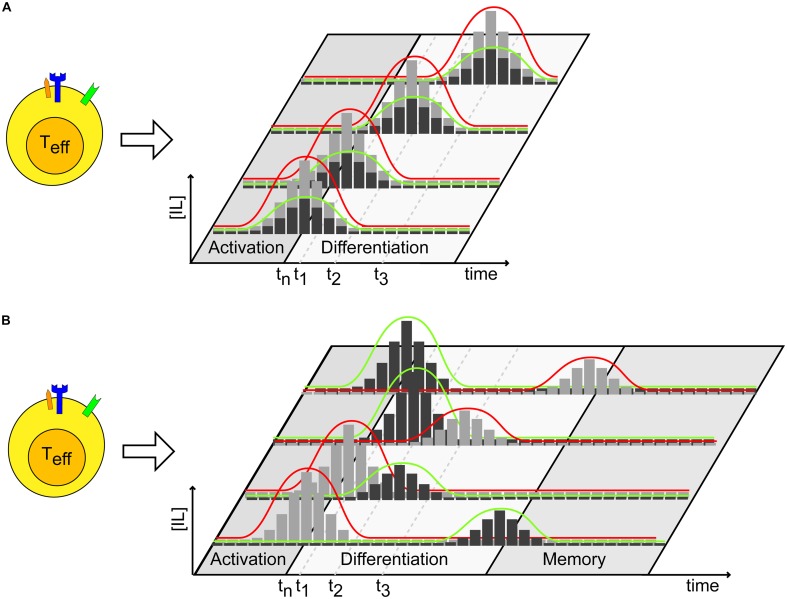
Cytokine dosage and administration timing impact T cell fate. **(A)** After activation, cytokines may be administered at different times and quantities (dosage) to investigate the effects on the dynamics of T cell differentiation. Black and gray bars represent a discrete administration of variable amounts of two different cytokines. t_n_ represents the time at which activation of naïve T-cells takes place. **(B)** Similar experiment as in **(A)** can be performed, where cytokines involved in different processes, such as differentiation and memory formation, are administered to T cells at different times and doses, or in a reversed order. Color legends are used as in **(A)**. t_n_ represents the time at which activation of naïve T-cells takes place.

By measuring the expression and abundance of T-bet (T_h_1) and GATA-3 (T_h_2) transcription factors, it is possible (*i*) to monitor whether a conditioned environment influences the appearance of a specific T cell subtype selection and, more importantly, (*ii*) to determine which cytokine threshold has a marked impact on the T cell differentiation process when using, for example, a dilution series of conditioned media (**Figure 2C**). These experiments may be performed by progressively varying the time of cytokine administration during prolonged TCR stimulation, to gather information about the specific time post-stimulation at which T cells are committed to a certain lineage. Specifically, we propose a scheme to conduct such experiments (**Figure 3**). The timing and dosage of action of specific cytokines, or conditioned media, can be varied by altering the moment of their addition in *in vitro* experiments (**Figure 3A**). By making dilution series, T cell differentiation can be followed as a function of time post-cytokine administration and of cytokine dosage. **Figure 3B** presents a further elaboration of the scheme, where variable scenarios of T cell activation, differentiation and memory may occur upon varying in time both the relative timing and dosage of the administered pattern of cytokines.

Hence, the aim is not only to identify which definite cytokine pattern stimulates the fate of T cell differentiation, but to determine quantitatively the specific threshold of cytokine dosage (concentration) and temporal constraints impinging the T cell decision lineage. These data may serve as input as well as validation for computer models that can temporally explore the requirement for cytokine dosage and *precise* timing of activity during the T cell differentiation process. Although genetic engineering is not a prerequisite in this scenario, the experiments presented in **Figures 2**, **3** may be of help to generate quantitative data that may serve as inputs for computer models. T cells expressing transcription factors tagged with fluorescent proteins may be investigated for their lineage status through time by quantitative, non-destructive measurement. Moreover, by using genetic engineering technologies that allow for modulation of the dosage of intracellular signaling components or transcription factors, control studies on these molecules with respect to the activation and differentiation of T cells may be investigated.

## Tipping the Balance Between Cytokine and TCR Dosage in Memory T Cell Development

Shaping the differentiation process of T cells after their activation is mainly dependent on two cues: (*i*) the magnitude of TCR activation and (*ii*) the pattern of cytokines available which, in turn, largely depends on the nature of the invading pathogen. In addition to the types of cytokines, their dosage (concentration) also impacts the speed and magnitude of differentiation into several types of effector and memory T cells. In an inflammatory environment, IL-2, IL-12, and type I IFN are required to unleash the full cytotoxic potential of activated CD8+ T cells. Importantly, low concentrations of these cytokines inhibit effector T cell formation while pushing the balance toward memory CD8+ T cell differentiation (Pennock et al., 2013).

Activation of naïve T cells and their differentiation into effector cells have been described as a function of antigen and MHC recognition, co-stimulation and survival signals. However, the relevance of several cues that stimulate CD4+ T cells to differentiate to the memory subtype, while deleting most effector cells, still remains debated. Mechanisms that have been proposed to be responsible for this switch include TCR-affinity (mainly antigen dissociation constants) (Kim et al., 2013), CD20 co-stimulation (Dooms and Abbas, 2006), and persistence of high expression levels of IL-2 (Dooms et al., 2007), IL-7 (Kondrack et al., 2003), and IL-15 (Purton et al., 2007) (**Figure 2D**). Notably, a crosstalk exists between TCR activation and cytokine signaling. The TCR signaling pathway that triggers activation of naïve T cells includes phosphorylation events through linker-adapter molecules such as the SH2-containing phosphoprotein SLP-76 (Jordan et al., 2003). CD4+ T cells carrying inactivated SLP-76 are unable to produce IL-7 and IL-15 upon TCR-mediated stimulation, despite IL-receptors being functional (Bushar et al., 2010). In line with this finding, artificial memory CD8+ T cells have been generated by treating isolated T cells with IL-7 and IL-15 (Cieri et al., 2013). Thus, tipping the balance between TCR and cytokine dosage influences the fate of memory T cells.

The development of CD4+ and CD8+ T cells is distinct, with CD4+ but not CD8+ T cells exhibiting lineage diversity. This diversity results in a variable distribution of memory cells within each lineage, with the distribution potentially varying due to the uncertainty in T cells that differentiate to the memory subtype to retain their lineage-differentiation traits (Gasper et al., 2014). Altogether, these open questions challenge the investigation of memory T cell formation. Although memory CD8+ T cells are stably maintained, the number of memory CD4+ T cells declines over time. Thus, the effector lineage of memory CD4+ T cells is furthermore difficult to track over time, as these display substantial diversity and under certain conditions can change their lineage commitment (Rosenblum et al., 2016). Furthermore, CD4+ T cells play a crucial role in the development of memory CD8+ T cells; it is however, not yet clear if the reverse also holds true (Williams et al., 2006).

## Interleukin and Cell Cycle-Mediated Crosstalk for Memory T Cell Formation

An increasing body of studies indicate that during T cell differentiation intracellular signaling cascades, such as the ones governing growth (Delgoffe et al., 2011; Hasan et al., 2015; Pollizzi et al., 2015, 2016; Zhang et al., 2016), metabolism (Almeida et al., 2016; Fischer et al., 2017; Walker and McKenzie, 2017; Yong et al., 2017) and cell cycle progression (Sicinska et al., 2003; Veiga-Fernandes and Rocha, 2004; Tejera et al., 2013; Gu et al., 2014; Rowell et al., 2014; Wells and Morawski, 2014; Delpoux et al., 2017; Patel et al., 2017), may impact the T cell proliferative capacity as well as the magnitude and duration of T cell-mediated responses.

Among cell cycle regulators, the cyclin-dependent kinase CDK2 and the CDK inhibitor p27^Kip1^ (in the following indicated as p27) appear to be crucial in T cell differentiation by shifting the balance between T cell activation and anergy. Specifically, CDK2-deficient CD4+ T cells exhibit an anergic state even if CD28 co-stimulation is present (Chunder et al., 2012); conversely, p27-deficient CD4+ T cells are activated upon TCR stimulation alone (Jatzek et al., 2012). These findings are also supported by early studies showing the involvement of p27 as anergy factor (Boussiotis et al., 2000; Li et al., 2006; Rudd, 2006). Furthermore, CD28 simulation – which leads to T cell proliferation – has been shown to diminish p27 levels through activation of the PKB/Akt pathway (Appleman et al., 2002) and of the SCF/Skp2 ubiquitin ligase responsible for p27 degradation (Appleman et al., 2006). Finally, p27 has been shown to regulate differentiation of both Th and Treg subtypes (Tsukiyama et al., 2001; Rowell et al., 2005; Singh et al., 2010; Iglesias et al., 2013; Jatzek et al., 2013).

This evidence indicates that p27 is a sensor of the T cell proliferation status, i.e., differentiation versus anergy (Wells and Morawski, 2014). We have recently proposed that the crucial tuning of p27 abundance and dynamics, which fails in a cancer scenario, is required for the correct progression throughout cell cycle (Barberis and Verbruggen, 2017). Here, we further propose that p27 acts as a timer to safeguard not only cellular proliferation, but also T cell differentiation by the tight regulation of its dosage and timing of activity. Interestingly, an early study pointed out a role for p27 in safeguarding against immune-mediated inflammation, although using glomerulonephritis (group of diseases that injure the part of the kidney that filters blood) as model system for inflammation (Ophascharoensuk et al., 1998). Thus, p27 is an ideal candidate to explore the *precise* timing of T cell differentiation by the MAmTOW methodology, with a variable dosage of the regulator possibly impinging on the appearance of a definite T cell lineage.

**FIGURE 4:**
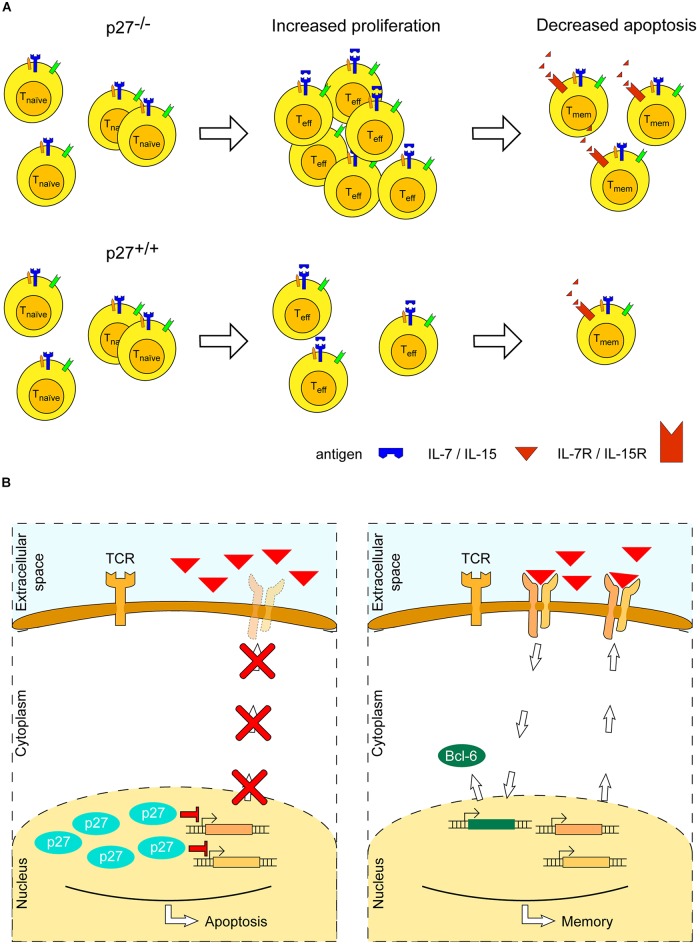
Role of the cyclin-dependent kinase inhibitor p27^Kip1^ in memory T cell formation. **(A)** Absence of p27^Kip1^ (p27) enhances expansion of T cells and survival of memory T cells: in the former, its absence boosts proliferation, whereas, in the latter, it decreases apoptosis. **(B)** p27 may act to inhibit expression of the receptor subunits of IL-7 and IL-15, which are required to promote memory T cell survival (left). When p27 is absent (right), consequent expression of IL-7 and IL-15 receptors may lead to the expression of anti-apoptotic proteins, such as Bcl-6, and to an enhanced memory T cell survival.

*In vivo* mice studies have shown that p27-deficiency enlarges the size of the thymus and spleen, and increases the amount of T cells in both naïve and activated states, by both increasing proliferation and reducing apoptosis (Fero et al., 1996; Nakayama et al., 1996). Further investigation has shown that the T cell proliferative potential was not the result of an increased expression of ‘survival’ cytokine receptors (CD127 and CD122, which sense IL-7 and IL-2, respectively), although it cannot be excluded that a greater autocrine and paracrine stimulation, due to an enlarged T cell number, may have enhanced the proliferative effect caused by p27-deficiency (Jatzek et al., 2012). No detectable difference in proliferation between wild type and p27-/- mice was observed in naïve T cells. However, surprisingly, memory CD4+ T cells exhibited increased levels of CD127 in p27-/- mice as compared to wild type and, concomitantly, a reduced apoptosis (Jatzek et al., 2012) (**Figure 4A**). This effect has been attributed to the binding of p27 to CDK, as mice expressing a variant of p27 lacking its CDK-binding domain accumulate CD4+ T cells as compared to wild type (Jatzek et al., 2012). Thus, pro-apoptotic and anti-proliferative functions of p27 regulate the differentiation and maintenance of memory CD4+ T cells, through its binding to CDK.

The evidence presented here highlights a putative double role of p27 in the development and differentiation of CD4+ T cells: (*i*) during the initial immune response, p27 inhibits proliferation of T cells upon CD28 co-stimulation, and (*ii*) p27 inhibits development of memory T cells by promoting apoptosis. The underlying molecular pathways are not entirely understood; however, a few mechanisms have been suggested (Jatzek et al., 2012): (*i*) an altered balance between the transcription factors T-bet, leading to T_h_1 terminal differentiation, and Bcl-6, leading to development of T_h_1 memory precursors (Oestreich et al., 2012), and (*ii*) a p27-dependent downregulation of the IL-7 and IL-2/IL-15 receptors [CD127 and CD122 (Meghnem et al., 2017), respectively], leading to a decreased expression of pro-survival/anti-apoptotic proteins, such as Bcl-2, that promote survival of memory T cells (Xue and Zhao, 2012) (**Figure 4B**).

Although a qualitative impact of p27 on CD4+ effector/memory T cells has been suggested, it remains unclear how its intracellular levels modulate these two routes. That is, the *precise* dosage at which p27 regulates the balance between T cell subtypes is at present unknown. Two unresolved questions are apparent: (*i*) Does a direct relation exist between the dosage of cytokines, specifically IL-7 and IL-15, and the p27 intracellular dosage in the formation and persistence of CD4+ memory T cells?, and (*ii*) Does a lower intracellular p27 dosage compensate for a reduced extracellular IL-7/IL-15 dosage?

These questions can be formed into hypotheses that are testable based on the experimental approaches that we have described. By generating T cell lines engineered such that they express inducible and tunable variants of p27 and/or the IL7/15 receptor, the influence of these components on T cell fate can be investigated. First, their expression can be measured as indicated in **Figure 1C**, with the genes of interest being under control of their endogenous promoter and their expression modulated by tetracycline induction (Barberis and Verbruggen, 2017). Second, the effects of cytokine pattern, timing and dosage on T cell proliferation may be tested as proposed in **Figures 2**, **3**. Here, expression (dosage) of p27 and/or of the IL7/IL15 receptor may be measured during T cell differentiation and memory T cell formation. Third, it can be determined how variation in the expression level of p27 and/or the IL7/IL15 receptors may impact a T cell becoming a memory T cell.

Altogether, the evidence presented here suggests that formation of memory T cells is a quantitative, but not a qualitative, process. The strategy described above would provide, for the first time, new insights about the interplay between the cell cycle machinery and T cell memory formation. The information gathered should form a solid basis for descriptive, quantitative models that accurately predict the role of dosage-mediated T cell differentiation and memory T cell formation.

Importantly, the integration of computer modeling and experimental validation requires considering the precise details of a biological process. This approach is particularly relevant for T cell differentiation and cell cycle control, for which complexity is required to realize timely responses upon a variety of input dynamic signals. The immune response thorough TCR signaling is distributed among many components, and computer models may be generated that analyze this complexity and investigate the specific contribution of individual components. Therefore, data-driven mathematical models of T cell differentiation may be developed that predict how a change in the stoichiometry of TCR signaling and cell cycle components impacts on T cell dynamics. Typically, the experimental data on which computer models are based are qualitative, i.e., interactions and regulatory activations/inhibitions among components. Because of the large number and complexity of the molecular interactions involved, computer simulations of well-parameterized mathematical models are crucial to design informative experiments. By incorporating in the models realistic dosage constraints – measured through experimental genetic engineering technologies – quantitative computer models may be generated. These data may converted to kinetic parameters, i.e., concentration of components, which represent an input for computer models. Computer models may in turn be employed to predict T cell dynamics by varying dosage, which can be then verified experimentally. Specifically, the copy number of TCR signaling and cell cycle components may be modulated – through tetracycline-based genetic engineering technologies – in mammalian cell lines to investigate paths that lead to various T phenotypes. Thus, a systematic exploration of model predictions by using quantitative data may lead to the identification of the precise dosage of components that are able to control T cell dynamics timely.

## Concluding Remarks

The innate and adaptive immune systems exhibit a dynamic crosstalk with, dogmatically, innate immunity taking the initial lead and gradually handing the defense over to its acquired counterpart. The entire process involves a multitude of cells of various origin and capabilities. Although the individual workings of many immune system’s components are well understood, it remains largely unknown how a robust immune response emerges as a resultant from the interplay of its components and how its fidelity and its kinetics are controlled.

Here, we have focused on the relevance of cytokine dosage for T cell activation and differentiation, pointing out the necessity to develop and implement experimental technologies that allow a high degree of control of intracellular signaling components in single-cell expression. The required experimental precision may be achieved by employing genetic engineering technologies such as CRISPR/Cas9, and the MAmTOW methodology that we have recently devised (Barberis and Verbruggen, 2017). The combination of these technologies can enable the investigation of the *precise* upper and lower expression boundaries of intracellular T cell signaling and transcription factors governing T cell activation/differentiation, and may help to determine their degree of control (if any) of the processes. The measurement of such boundaries for these components will then allow for *in silico* mathematical models to comprehend and predict how these shape the activation/differentiation response of T cells, and prevent an unwanted (premature or delayed) immune response. Indeed, computer models of T cell differentiation may be able to test *in silico* the effect of a characteristic dosage of specific components on the timing of a T cell response.

Interestingly, other types of balance may be investigated as well. For example, the intracellular balance between pro-apoptotic and anti-apoptotic signals may be potentially involved in the differentiation to memory T cells. By reducing or lengthening the lifespan of effector T cells, the balance of these signals allows for differentiation to memory T cells (Jay et al., 2013). In this scenario, the relevance of the quantitative balance between pro- versus anti-apoptotic signals, such as Bim and Bcl-6, respectively, may be investigated. For memory T cells, another balance may be investigated. CD4+ and CD8+ T cells can develop into two subtypes of memory cells: TEM (T effector memory) and TCM (T central memory) cells. The difference between these two memory T cell populations predominantly resides in their localization, which is dictated by the absence or presence of distinct sets of chemokine receptors and in the produced cytokine pattern upon TCR re-stimulation (Pepper and Jenkins, 2011; Rosenblum et al., 2016). Extensive investigations have been mounted for memory CD4+ T cells to investigate whether TEM/TCM formation may be lineage-dependent. However, no clear and unambiguous relation has been revealed yet (Pepper and Jenkins, 2011).

Finally, the integration between computer models and experimentation has the potential to (*i*) identify new relationships among different phenotypes of differentiated T cells, (*ii*) elucidate the molecular mechanisms leading to the various T differentiation lineages, and (*iii*) suggest specific administration schemes (i.e., input patterns, dosage and *precise* timing) that may be used in immune therapy for modulating the balance of T cell phenotypes. Recently, we and others have illustrated through an analysis of logical models of T cell differentiation that lineage is a function of both input patterns and their dosage (Martinez-Sanchez et al., 2018; Puniya et al., 2018). In particular, our model is able to predict novel, non-canonical phenotypes of differentiated T cells, which may play a role in the overall balance of the immune response (Puniya et al., 2018).

## Future Perspectives: Cell Cycle Control of Autoimmunity

The integrity of the immune system can be compromised by both intrinsic and extrinsic factors. This may then lead to a loss-of-specificity toward the pathogen, and consequent destruction of body’s cells. This process, called autoimmunity, may lead to a plethora of pathologies and symptoms, widely varying in their cause, onset and severity. The emergence of autoimmunity is a complex process involving many and different types of cells, effectors, antigens, and both cell-to-cell and molecular interactions of various nature. For example, it can arise due to (epi)genetic predisposition, or to cross-reactivity of antibodies against invading pathogens with self antigens. Whereas in restricted cases the provoking stimulus for autoimmunity is known, in others it seems to arise spontaneously with no rationale mechanism being uncovered.

Although, the human immune system functions similarly in most individuals, it is not yet understood how inter-person differences arise. We hypothesize that a random variability in intra-cellular components of the immune system may translate in its variability in function; this may be responsible for an accidental cellular proliferation upon recognition of a self antigen, potentially leading to an autoimmune response that would impact the overall system’s robustness. In this context, we speculate that the consequence of a spontaneous occurrence of autoimmunity may be cell cycle related. Cell cycle control plays a central role in the decision of whether T cells proliferate upon stimulation, or whether they become unresponsive, insensitive, and anti-proliferative, with CDK2 and p27 exerting a pivotal role in the balance of proliferation versus anergy (Rowell et al., 2005, 2014; Wells and Morawski, 2014). For example, to transit to a proliferative state, T cells need to overcome arrest in the G1 phase of the cell cycle mediated by p27 after co-stimulation (Rudd, 2006). An abundance of p27 in a T cell recognizing an antigen against the self may thus critically limit its proliferation and prevent an autoimmune response. Importantly, p27 inhibits systemic autoimmunity through the control of activity and differentiation of Treg cells (Iglesias et al., 2013), and it has been recognized as a sensor of the T cell proliferation status, specifically as anergy factor (Boussiotis et al., 2000; Li et al., 2006; Rudd, 2006; Wells and Morawski, 2014).

**FIGURE 5:**
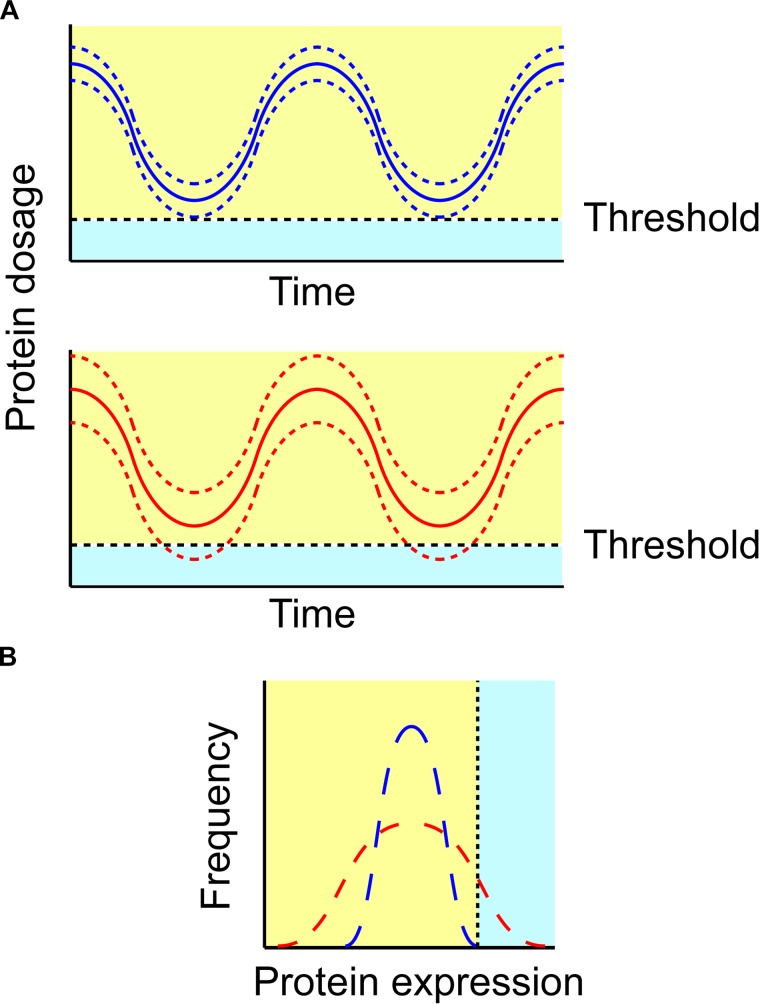
Variability in protein dosage impacts system’s robustness. **(A)** Protein expression may oscillate over time. If the variability in protein expression between cells increases, some cells may express a protein beyond the boundaries of robustness (light blue area; lower). Solid (blue and red) lines indicate the mean of protein expression in a cell population over time, whereas dashed lines indicate cell-to-cell variability. **(B)** Protein expression should be assessed at the single-cell level in order to investigate the impact of protein variability. Both (blue and red) distributions reveal the same mean, however, the variability of the red protein distribution may lead some cells to express it out of the cell’s robustness boundaries.

Within a defined cell population the expression of all proteins, including p27, may exhibit considerable variability. This concept is presented in **Figure 5A**, where expression of a protein (supposedly p27) in two different cells - where p27 is indicated by blue and red color, respectively for the two different cells - is oscillating over time. The two cells have a similar periodicity and mean expression levels of p27, but they differ in p27 expression variability, which is indicated with lowest and highest borders (plotted as dotted lines). In **Figure 5B**, a hypothetical frequency histogram is plotted for p27 levels in both cells.

Let us hypothesize this protein being p27. The spatiotemporal regulation of p27 has been investigated in detail experimentally, with p27 exhibiting an oscillatory behavior throughout cell cycle progression (Starostina and Kipreos, 2012). Specifically, p27 levels (*i*) are high in the G1 phase of the cell cycle, to inhibit an earlier cyclin/Cdk2 activity and prevent an untimely entry into S phase, and (*ii*) drop at the G1/S transition, to release cyclin/Cdk2 inhibition and allowing DNA replication to start (Starostina and Kipreos, 2012). Even though a cell may not divide, the protein concentration oscillates, due to periodical protein turnover and, likely, also due to stochastic gene transcription. The variability in p27 levels due to the latter aspect differs between the two cells, being larger for the cell with p27 drawn in red color. Now, let us suppose that a definite threshold of activation exists, which would need to be reached before p27 level is sufficiently low to allow cell cycle exit. This condition, under the given circumstances, is never met for the blue protein; hence, the cell will remain in a state of quiescence. Conversely, the red protein may occasionally reach the definite threshold of activation during its periodic cycle, and will exit the cell cycle, due to the variability in stochastic gene transcription.

Translating this scenario to the role of p27 in the immune system, the two variable p27 protein (blue and red) levels may be exhibited by two T cells. Let us suppose that the definite threshold represents a critical p27 level at which the stimulatory capacity toward the self antigens is sufficient to provoke an unwanted T cell proliferation. The T cell carrying the blue p27 protein will never reach this condition, as its p27 level will be never sufficiently low to cross the threshold. This scenario may be characteristic of a healthy individual. A genuine antigen, however, will raise this threshold enabling T cell proliferation. That is, the T cell carrying the red p27 protein – that exhibits a sufficient oscillation to cross the threshold at some time points – may prove potentially vulnerable to proliferation upon stimulation by self antigens. This scenario may be characteristic of an individual with an immune disease. Although both T cells will become anergic in most cases when self antigens are encountered, the cell with the red p27 protein can proliferate under certain conditions, thus potentially stimulating an immune response against the self.

As described here for intracellular T cell signaling components, genetic engineering technologies can be employed to systematically measure upper and lower boundaries of gene and/or protein dosage, to gain understanding about their impact on autoimmunity. Thus, the gene expression ceiling may be related to whether immune cells would be activated by a weak stimulus or a self antigen. Practically, a fluorescent protein controlled by the promoter of an inflammatory gene may be used as readout for the expression level of the target protein, or the cells may be checked for the expression of specific surface markers. An example of an inflammatory gene that may be tested is the transcription factor FOXP3. Maintenance of an appropriate expression level of FOXP3 leads to the differentiation and maturation of Treg (regulatory) cells (Hori et al., 2003), which play important roles in immunological tolerance (Xiao et al., 2013). Mice and humans with a loss of FOXP3 function develop autoimmunity and inflammatory disease. Activated T cells can transiently upregulate FOXP3 levels (Gavin et al., 2006), and activated T cells carrying high and stable expression levels of FOXP3 can become immunosuppressive (Wang et al., 2007). Interestingly, crosstalk may be realized to maintain FOXP3 levels, positively mediated by IL-2 (Fontenot et al., 2005), and negatively regulating its stability and activity by CDK2 (Morawski et al., 2013).

Altogether, the evidence presented in this study suggests that an interplay of cell cycle with T cell-specific factors may modulate T cell proliferation. A definite dosage of these factors may determine the outcome of the delicate balance between proliferation and anergy, and the same may apply to the balance between immune and autoimmune response. Thus, the MAmTOW methodology that we have recently envisioned may be of help to investigate these delicate balances, therefore to explore the robustness of the immune system.

## Author Contributions

MB conceived the idea, designed the study, provided scientific leadership, supervised the study, and wrote the manuscript, with contribution from PV for the experimental parts and from TH for the modeling part elaborated in Section “Cytokine Pattern and Dosage Determine T Cell Differentiation”. PV helped with the final layout of the figures.

## Conflict of Interest Statement

The authors declare that the research was conducted in the absence of any commercial or financial relationships that could be construed as a potential conflict of interest.
